# Clinical Characteristics and Development of Complications Differ Between Adult-Onset and Child–Adolescent-Onset Type 1 Diabetes: A Report From a Tertiary Medical Center in Türkiye

**DOI:** 10.1155/jdr/8860118

**Published:** 2025-04-09

**Authors:** Ramazan Çakmak, Özge Telci Çaklılı, Ayşe Merve Ok, Ümmü Mutlu, Göktuğ Sarıbeyliler, Vefa Seferova Nasifova, Ersel Bilgin, Aylin Çoşkun, Damla Yenersu Guzey, Utku Erdem Soyaltin, Servet Yüce, Hülya Hacışahinoğulları, Gülşah Yenidünya Yalın, Özlem Soyluk Selçukbiricik, Nurdan Gül, Ayşe Kubat Üzüm, Kubilay Karşıdağ, Nevin Dinççağ, Mehmet Temel Yılmaz, Ilhan Satman

**Affiliations:** ^1^Department of Internal Medicine and Division of Endocrinology and Metabolic Diseases, Istanbul Health and Technology University, Istanbul, Türkiye; ^2^Department of Internal Medicine and Division of Endocrinology and Metabolic Diseases, Istanbul Faculty of Medicine, Istanbul University, Istanbul, Türkiye; ^3^Department of Internal Medicine, Istanbul Faculty of Medicine, Istanbul University, Istanbul, Türkiye; ^4^Istanbul Faculty of Medicine, Istanbul University, Istanbul, Türkiye; ^5^Department of Internal Medicine and Division of Endocrinology and Metabolic Diseases, Basaksehir Cam and Sakura City Hospital, Istanbul, Türkiye; ^6^Department of Public Health and Biostatistics, Istanbul Faculty of Medicine, Istanbul, Türkiye

**Keywords:** adult-onset Type 1 diabetes, child–adolescent-onset Type 1 diabetes, C-peptide, diabetic ketoacidosis, microvascular complications

## Abstract

**Background and Aims:** The age-at-onset is of great importance in the heterogeneity of Type 1 diabetes mellitus (T1DM). This study was designed to define clinical and laboratory differences between child–adolescent-onset and adult-onset T1DM at presentation and during follow-up and determine the predicting factors for developing microvascular and macrovascular complications.

**Material and Methods:** This retrospective observational study evaluated T1DM patients who were followed in the diabetes outpatient clinic between January 1, 2000, and December 31, 2019.

**Results:** The study cohort included 490 individuals with T1DM (54.3% female, 58.8% adult-onset, and median follow-up: 5 years). In the adult-onset group, baseline C-peptide and GADA prevalence were higher, whereas presentation with ketoacidosis was 2.3-fold lower compared to the child–adolescent-onset group (*p* < 0.001). During follow-up, the adult-onset group had a 2.4-fold higher overweight/obesity (*p* < 0.001) and 1.7-fold higher dyslipidemia/hyperlipidemia (*p* = 0.002) than the child–adolescent-onset group. In multivariate analysis, fasting glucose (*p* = 0.024) in adult-onset, dyslipidemia/hyperlipidemia (*p* = 0.037) in child–adolescent-onset, and diabetes duration (*p* = 0.008 and *p* = 0.007) and hypertension (*p* = 0.001 and *p* = 0.01) in both groups were associated with increased risk of microvascular complications, whereas age-at-onset (*p* = 0.024), dyslipidemia/hyperlipidemia (*p* = 0.03), nephropathy (*p* = 0.003), and neuropathy (*p* = 0.001) in adult-onset and age (*p* = 0.002) and triglycerides (*p* = 0.013) in child–adolescent-onset groups were associated with increased risk of macrovascular complications. The cutoff C-peptide levels at baseline predicted microvascular complications in the whole cohort and adult-onset group were defined as 0.383 ng/mL (*p* < 0.001) and 0.41 ng/mL (*p* = 0.001), respectively. In the Kaplan–Meier analysis, C-peptide (< 0.383 ng/mL) but not age-at-onset predicted future development of microvascular and macrovascular complications (*p* = 0.003 and *p* = 0.032).

**Conclusion:** Clinical presentation and prognosis differ in adult-onset and child–adolescent-onset T1DM. Low initial C-peptide may predict the development of microvascular and macrovascular complications.

## 1. Introduction

Type 1 diabetes mellitus (T1DM) is a chronic autoimmune disease characterized by insulin deficiency and hyperglycemia due to the destruction of pancreatic *β*-cells. Although T1DM is one of the most common endocrine diseases in childhood, it may onset at any age and represents approximately 5%–10% of diabetes cases [1]. Recent data suggest that more than half of cases of T1DM begin in adulthood [2]. T1DM represents clinically quite heterogeneous subtypes. To explain this heterogeneity, different endotypes of T1DM have been defined [3, 4]. T1DM endotypes underscore the importance of individualized care. However, our information about the presentation and clinical course of the disease comes mostly from publications on childhood T1DM, and there is little data on adult-onset types, limited to European and US populations where the incidence of childhood diabetes is relatively higher.

Despite recent advances in diabetes technology and management, many patients with T1DM develop microvascular and macrovascular complications that can result in significant morbidity and mortality [2, 3]. Our knowledge about the progression of complications is also limited to childhood-onset T1DM, and both epidemiological and clinical studies in adult-onset T1DM are scarce. Furthermore, the link between microvascular and macrovascular complications is not clear enough. In this study, we evaluated the difference in mode of onset, clinical features, and laboratory findings between adult-onset and child–adolescent-onset T1DM and identified factors predicting the risk of microvascular and macrovascular complications.

## 2. Materials and Methods

This retrospective observational study evaluated T1DM patients who were followed in the diabetes outpatient clinic between January 1, 2000, and December 31, 2019. It was approved by the local ethical review board. Due to the retrospective design of the study, patients' informed consent was waived.

### 2.1. Patients and Data

Diabetes was diagnosed according to national and international guidelines [5, 6]. Inclusion criteria were as follows: (1) diagnosed with clinical T1DM and (2) followed by our outpatient team for at least 1 year. Those who did not require insulin from the beginning were excluded from the analysis. Child–adolescent-onset T1DM patients were followed by pediatric endocrinologists until the age of 18 and then transferred to our unit. Adult-onset T1DM patients were followed in our endocrine or internal medicine (in consultation with endocrinology) outpatient clinics. The baseline data of patients who did not initially apply to the endocrine clinic, especially the child–adolescent group, were obtained from electronic health records (EHRs) and medical reports.

Arterial hypertension was defined in accordance with the World Health Organization/International Society of Hypertension guidelines [7] and dyslipidemia/hyperlipidemia as per the National Cholesterol Education Program Adult Treatment Panel III criteria [8]. Insulin dose-adjusted glycated hemoglobin A1c (IDA-HbA1c) was calculated by the equation: “*HbA*1*c* (%) + [4 × *insulin* *dose* (*units*/*kg*/24 *h*)]” [9]. The estimated glomerular filtration rate (eGFR) was calculated by the chronic kidney disease (CKD)-EPI formula [10].

All patients were screened for islet autoantibodies (mostly islet-cell cytoplasmic-ICA and glutamic acid decarboxylase (GADA)) and also for autoantibodies of coexisting autoimmune diseases against endomysium and/or transglutaminase for celiac disease, thyroid peroxidase (TPOA) and thyroid stimulating hormone receptor for autoimmune thyroid disease, and gastric parietal cell (GPCA) for pernicious anemia. Fasting plasma glucose (FPG), HbA1c, and serum creatinine; total, low-, and high-density lipoprotein cholesterol (total-C, LDL-C, and HDL-C); and triglycerides (TG), urine albumin-to-creatinine ratio (UACR), and C-peptide levels were checked in all patients at the first visit and thereafter every 3–12 months. Demographic features, diabetes complications, coexistent and autoimmune diseases, laboratory findings, and treatment modalities were obtained from EHRs.

### 2.2. Definitions of Diabetic Complications

While retinopathy, neuropathy, and nephropathy were grouped in microvascular, coronary artery disease (CAD), cerebrovascular accident (CVA), peripheral artery disease (PAD), and diabetic foot ulcer (DFU) were grouped in macrovascular diabetic complications. All patients were screened for chronic complications at presentation and periodically according to the national clinical practice guidelines [6]. Proliferative and/or nonproliferative retinal findings detected by experienced ophthalmologists were defined as diabetic retinopathy [11]. Patients who had neuropathic signs and/or symptoms were evaluated for diabetic neuropathy by quantitative sensorial and electrochemical skin conductance tests; if needed, electromyography was ordered after excluding other etiologies [12]. Established diabetic nephropathy (CKD) was defined by the presence of persistently declined eGFR (< 60 mL/min/1.73 m^2^) and/or elevated UACR (>30 mg/g) for more than 3 months in accordance with KDOQI guidelines [13]. CAD was defined as either the presence or history of stable angina pectoris, acute coronary syndrome, or revascularization history.

### 2.3. Measurements

Biochemical analysis was performed from serum samples by an electrochemiluminescence method (Beckman Coulter Unicel DXI 800 Autoanalyzer, Brea, CA, United States). Serum hormone levels were analyzed by an immunodiagnostic system (Siemens, Advia Centaur XP, Germany). HbA1c levels were measured by the turbidimetric immunoinhibiting method (Beckman Coulter Au480 model automated analyzer).

### 2.4. Statistical Analysis

The Statistical Package for the Social Sciences program v21.0 (SPSS, IBM, Armonk, NY, United States) was used for the analysis of the data. Descriptive variables were expressed as mean ± standard deviation (SD) or median and interquartile range (IQR), and categorical variables as numbers and percentages. Chi-square and Fisher's exact tests were used to compare categorical variables. Normality distribution was checked by the Kolmogorov–Smirnov test. Parametric and nonparametric data were assessed with Student's *t* and Mann–Whitney *U* tests, respectively. Correlation analysis was performed by Pearson's or Spearman's methods according to normality distribution. Multivariate analysis was used by the forward logistic regression model; while microvascular and macrovascular complications were chosen as dependent variables, parameters whose *p* < 0.20 in univariate analysis were included as independent variables. Receiver operating characteristic (ROC) analysis was used to calculate the cutoff value of relevant biomarkers such as C-peptide and HbA1c, indicating the development of microvascular and macrovascular complications. Sensitivity analysis was performed to determine whether the subgroups with available or unavailable antibody results have similar characteristics. We constructed three logistic regression models to assess the prevalence of complications while controlling for potential confounders: Model-1 unadjusted, Model-2 to adjust for age and diabetes duration, and Model-3 to further adjust for smoking and alcohol consumption. Kaplan–Meier with the log-rank method was applied for survival analysis of the development of microvascular and macrovascular complications according to the relevant biomarkers. A *p* value <0.05 was considered statistically significant.

## 3. Results

The study cohort consisted of 490 individuals (54.3% women); of these, 41.2% were diagnosed with T1DM before the age of 18 (child–adolescent-onset) and 58.8% at the age of 18 or later (adult-onset). Patients were followed for a median of 5 years. Baseline demographic and laboratory data of the study groups are shown in Table 1. There was no significant difference between the two groups in terms of gender, duration of follow-up, and family history of diabetes. Tobacco use was more common in the adult-onset group (*p* = 0.001). While the presentation with diabetic ketoacidosis (DKA) was 34.4% in the child–adolescent-onset group, this rate was 15.2% in the adult-onset group (*p* < 0.001). Additionally, GADA prevalence (65.2% vs. 35%; *p* < 0.001) and baseline C-peptide levels were higher (*p* < 0.001), and eGFR was slightly lower (*p* = 0.001) in the adult-onset group. There was no significant difference in the prevalence of ICA, initial FPG and HbA1c levels, daily insulin doses, and fasting lipid profiles between the two groups.

The prevalence of microvascular and macrovascular complications and comorbidities in the groups is presented in Table 2. During the follow-up period, 34% of cases in the adult-onset and 37% of cases in the child–adolescent-onset groups developed at least one microvascular complication with no significant difference. The rate of developing at least one macrovascular complication was 8% in the adult-onset and 5% in the child–adolescent-onset groups. The individual distribution of macrovascular complications and the prevalence of hypertension did not differ between the two groups, whereas dyslipidemia/hyperlipidemia, overweight, and obesity were more common in the adult-onset group (*p* = 0.002, *p* = 0.003, and *p* < 0.001, respectively). At follow-up, 41.5% of the adult-onset and 43% of the child–adolescent-onset groups had at least one autoimmune disease in addition to T1DM. Most of these were women with autoimmune thyroid diseases (mainly Hashimoto's thyroiditis). Furthermore, metformin (*p* < 0.001), acetylsalicylic acid (ASA) (*p* = 0.007), and statins (*p* = 0.004) were more frequently used in the adult-onset group.

Baseline autoantibody results were not available in some cases, particularly in the child–adolescent-onset group. Therefore, we performed a sensitivity analysis between subgroups with and without autoantibody results available and compared the child–adolescent- and adult-onset subgroups. The adult-onset subgroup with available antibody results was older, had a shorter diabetes duration, a higher body mass index (BMI), fewer cases presented with DKA, a higher prevalence of GADA and overweight/obesity, a higher initial C-peptide, and a lower eGFR compared to the child–adolescent-onset subgroup. Not surprisingly, similar results were observed except for smoking, TG, and HDL-C when comparing the adult-onset and child–adolescent-onset subgroups without autoantibody results, in which the adult-onset group had a significantly higher smoking rate, higher median TG, and lower median HDL-C levels (Table S1). We also performed another sensitivity analysis according to the presence or absence of antibody results in the child–adolescent- and adult-onset groups separately. In the child–adolescent-onset group, those with antibody results were younger and had a shorter duration of diabetes and lower prevalence of retinopathy during follow-up, compared with the subgroup without antibody results. While similar significant differences were found in the adult-onset group, the subgroup with antibody results additionally had higher initial C-peptide levels and lower initial insulin doses (Table S2).

**Figure 1:**
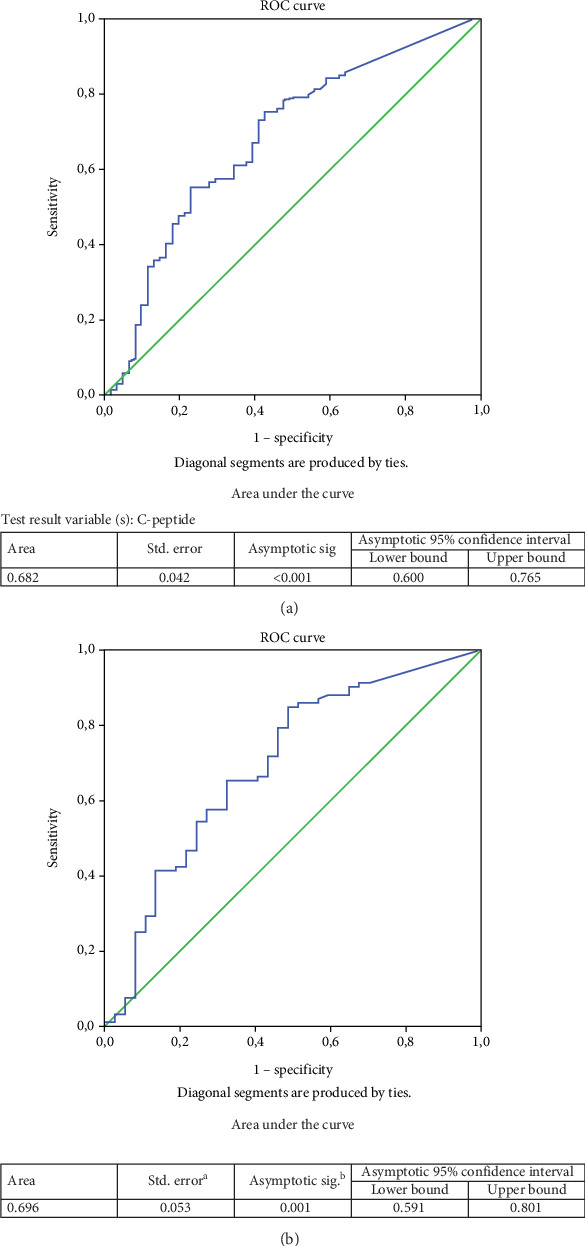
The best cutoff for baseline C-peptide levels indicating future development of microvascular complications (a) in the whole group and (b) in the adult-onset T1DM. ROC analysis (a) identified the best baseline C-peptide cutoff value for the development of microvascular complications (a) in the entire T1DM group as 0.383 ng/mL with optimal sensitivity (57%) and specificity (72%) and (b) the adult-onset T1DM group as 0.41 ng/mL with optimal sensitivity (65.2%) and specificity (64.9%). However, ROC analysis could not identify any optimal C-peptide cutoff value in the child–adolescent-onset T1DM group.

The best cutoff for baseline C-peptide to predict microvascular complications was determined by ROC analysis (Figure 1). While in the whole study population, a baseline C-peptide of 0.383 ng/mL predicted microvascular complications with 57% sensitivity and 72% specificity (area under the curve (AUC): 0.682; 95% confidence interval (CI): 0.600–0.765; *p* < 0.001); in the adult-onset group, a baseline C-peptide of 0.41 ng/mL predicted microvascular complications with 65.2% sensitivity and 64.9% specificity (AUC: 0.696; 95% CI: 0.591–0.801; *p* < 0.001). No significant baseline C-peptide cutoff could be defined to indicate the development of microvascular complications in the child–adolescent-onset group. On the other hand, because the number of cases with macrovascular complications was low in both groups, no cutoff baseline C-peptide level could be determined that would indicate the development of macrovascular complications. Similarly, a baseline HbA1c of 8.55% predicted microvascular complications with 65.5% sensitivity and 53.8% specificity in the adult-onset group (AUC: 0.607, 95% CI: 0.537–0.678; *p* = 0.005) (data not shown).

Table 3 shows the univariate and multivariate analyses of microvascular complications in child–adolescent- and adult-onset groups. In the younger-onset group, the mean age of patients who developed microvascular complications was older; the duration of diabetes was longer; baseline total-C and LDL-C levels were higher; and the prevalence of hypertension, dyslipidemia/hyperlipidemia, and any macrovascular complication, especially DFU, was higher compared to those without microvascular complications. In multivariate analysis, duration of diabetes (odds ratio (OR): 1.2; 95% CI: 1.05–1.37; *p* = 0.007), hypertension (OR: 18.6; 95% CI: 1.9–184.6; 95%; *p* = 0.010), and dyslipidemia/hyperlipidemia (OR: 7.1; 95% CI: 1.2–45.2; *p* = 0.037) were significantly associated with the risk of microvascular complications.

According to the univariate analysis, adult-onset patients with microvascular complications were older and had longer duration of diabetes and mean follow-up; higher median FPG, HbA1c, and TG levels; and lower C-peptide levels at baseline. In addition, hypertension, dyslipidemia/hyperlipidemia, CAD, and DFU were more common compared to those without microvascular complications. In multivariate analysis, duration of diabetes (OR: 1.08; 95% CI: 1.02–1.13; *p* = 0.008), baseline FPG levels (OR: 1.005; 95% CI: 1.001–1.009; *p* = 0.024), and hypertension (OR: 3.9; 95% CI: 1.4–10.8; *p* = 0.010) were significantly associated with the risk of microvascular complications.

Table 4 shows the univariate and multivariate analyses of factors associated with the risk of macrovascular complications in child–adolescent-onset and adult-onset T1DM groups. In univariate analyses, cases developing macrovascular complications in the younger-onset group were, on average, older and had a longer duration of diabetes; higher LDL-C and TG levels at baseline; and a higher prevalence of hypertension, dyslipidemia/hyperlipidemia, and any microvascular complication, particularly retinopathy and neuropathy, compared with those without macrovascular complications. In multivariate analysis, only age (OR: 1.2; 95% CI: 1.05–1.26; *p* = 0.002) and baseline TG levels (OR: 1.01; 95% CI: 1.002–1.017; *p* = 0.013) were significantly associated with the risk of macrovascular complications in this group. In contrast, univariate analysis in the adult-onset group showed that those who developed macrovascular complications were older and had longer duration of diabetes; higher age-at-onset; and higher prevalence of ICA, hypertension, dyslipidemia/hyperlipidemia, and any microvascular complication; in addition, elevated UACR (> 30 mg/g), declined eGFR (< 60 mL/min), retinopathy, and neuropathy were more common in those who developed macrovascular complications at follow-up. In multivariate analysis, age-at-onset of diabetes (OR: 1.07; 95% CI: 1.01–1.13; *p* = 0.024), dyslipidemia/hyperlipidemia (OR: 5.7; 95% CI: 1.16–27.9; *p* = 0.030), declined eGFR (OR: 15.4; 95% CI: 2.6–92.2; *p* = 0.003), and neuropathy (OR: 13.2; 95% CI: 2.8–62.4; *p* = 0.001) were identified as factors significantly associated with the risk of macrovascular complications.

A comparison of the child–adolescent- and adult-onset T1DM subgroups who developed microvascular complications showed that the adult-onset subgroup had an older age, shorter diabetes duration, longer follow-up, higher BMI, less presentation with DKA, higher frequency of GADA, higher HbA1c at disease onset, lower HDL-C, and higher TG levels. In addition, compared to the younger-onset group, more patients in this cohort had an HbA1c above the IDA-HbA1c at baseline. Moreover, this subgroup was more likely to develop dyslipidemia/hyperlipidemia and overweight/obesity over time (Table S3). A similar comparison for those who developed macrovascular complications showed that the adult-onset T1DM subgroup had older age, shorter diabetes duration, higher BMI, and higher prevalence of at least one autoantibody-positive at presentation. Additionally, the prevalence of overweight/obesity was higher in this subgroup (Table S4).

We also compared child–adolescent-onset and adult-onset T1DM groups with respect to complications, comorbidities, and concomitant medications after controlling for confounding factors (Table 5). In the unadjusted analysis (Model-1), the adult-onset T1DM group had a significantly higher prevalence of dyslipidemia/hyperlipidemia (*p* = 0.002), overweight (*p* = 0.003), and obesity (*p* < 0.001) as well as higher use of concomitant medications such as metformin (*p* < 0.001), ASA (*p* = 0.007), and statin (*p* = 0.004). However, there was no difference in microvascular and macrovascular complications between the two groups. After adjustment for age and diabetes duration (Model-2), the difference between comorbidities and medications was no longer significant. However, the odds of developing any macrovascular complication were significantly higher in the adult-onset group (*p* = 0.007), and most of the risk appeared to be due to the development of CAD (*p* < 0.001). These remained significant after further adjustment for lifestyle factors such as smoking and alcohol consumption (overall macrovascular complications: *p* = 0.009 and CAD: *p* < 0.001) (Model-3).

**Figure 2:**
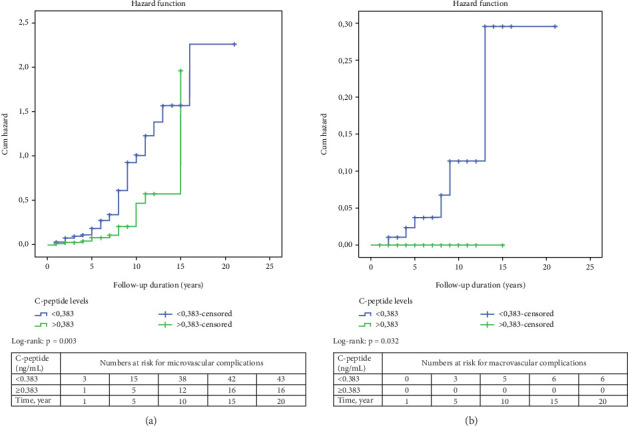
The Kaplan–Meier survival analysis for the development of (a) microvascular and (b) macrovascular complications according to baseline C-peptide levels. T1DM patients with baseline C-peptide value less than 0.383 ng/mL had a higher risk of developing (a) microvascular and (b) macrovascular complications.

The results of the Kaplan–Meier survival analysis are shown in Figure 2. Accordingly, the risk of developing microvascular and macrovascular complications was higher in patients with C-peptide levels below 0.383 ng/mL compared to those with higher C-peptide levels at presentation (log-rank *p* = 0.003 and *p* = 0.032, respectively). Meanwhile, survival analysis by age-at-onset of diabetes (< 18 years vs. ≥ 18 years) could not detect a significant difference in the risk of developing microvascular or macrovascular complications.

## 4. Discussion

In this single-center retrospective observational cohort study, we compared adult-onset and child–adolescent-onset T1DM groups by evaluating baseline and follow-up findings. We also examined the factors predicting the development of microvascular and macrovascular complications during the follow-up period (2000–2019). Approximately 60% of the study cohort had adult-onset T1DM. Except for the prevalence of the islet autoantibodies, the presentation with DKA, and the initial C-peptide levels, the clinical and laboratory data and proportion of complications were similar in the two groups.

The age-at-onset of T1DM is determined by the intensity of the *β*-cell destructive process, which is modulated by genetic and environmental factors [2, 14]. T1DM is traditionally considered a disease of childhood. However, adults with T1DM increased substantially over the past few decades. According to a recent systematic review, the incidence of adult-onset T1DM in countries/regions was higher in the Nordic countries. However, data were lacking in low/middle-income countries [15]. According to IDF and others [16–18], T1DM is increasing in young adults. In the Australian national database, T1DM was more common in people over 60 years (33.4%) compared to children and adolescents (11.1%) [19]. The life expectancy of people with T1DM has increased since the 1990s, along with a considerable decrease in mortality and disability-adjusted life years (DALYs). Between 1990 and 2019, the global age-standardized prevalence of T1DM in older adults tended to increase, while mortality and age-standardized DALYs tended to decrease [20].

IDF and others reported that both childhood and adult-onset T1DM are more common in men [14–16]. Although the incidence of T1DM in the elderly is generally considered similar among women and men, studies from Sweden and Spain reported higher incidence in women [17]. In contrast, both child–adolescent-onset and adult-onset T1DM occurred at similar rates among men and women in our cohort.

Another striking finding of our study was the high prevalence of family history of diabetes (concerning first- and second-degree relatives) in both child–adolescent and adult-onset T1DM (59% vs. 64%). In a Polish study, T1DM was detected in 5.5% of first-degree relatives of patients with T1DM [21]. In a population-based Swedish study, children with T1DM were more likely to have parents with T2DM compared to children without diabetes [22]. Moreover, these children were more likely to be overweight/obese. In a retrospective study from China (2018–2022), 28.2% of patients with T1DM diagnosed before 14 years had a family history of T2DM [23]. In line with the literature, our cases with a family history of diabetes had mostly relatives with T2DM, while those with T1DM were quite low (8.2%). The higher prevalence of family diabetes in our cohort may be related to the high rate of consanguineous marriages in the country [24].

Recently, several endotypes have been defined to explain the clinical and laboratory heterogeneity of T1DM. While C-peptide levels are lower, presentation with DKA is more frequent, and autoantibodies are more common in child–adolescent-onset T1DM; cardiovascular risk factors are more prominent in adult-onset T1DM [4]. In the INNODIA study and the T1DM change clinic registry, the presentation of DKA increased with decreasing age [25, 26]. In our study, the presentation with DKA was 2.3-fold higher in child–adolescent-onset T1DM than in the adult-onset group. In the adult-onset group, the prevalence of dyslipidemia/hyperlipidemia was 1.7-fold, and the prevalence of overweight/obesity was 1.4-fold higher than in the child–adolescent-onset T1DM group, consistent with the endotype theory.

Among the islet autoantibodies, GADA and ICA are most commonly found in new-onset T1DM patients. IA-2A and zinc transporter 8 antibody-ZnT8A are less common, being detected in approximately 60% of new-onset T1DM subjects. While GADA is present more frequently in adult-onset T1DM, IAA is present in 50% of children with new-onset T1DM. GADA can be detected during the first few years after diagnosis, whereas others disappear more rapidly [27, 28]. ICA is not a single antibody but reflects multiple autoantibodies against *β*-cell antigens as immunofluorescence in pancreatic islets [29]. In the INNODIA study, 96.9% of T1DM cases were positive for ≥ 1 autoantibodies [25]. However, another study showed a lower frequency of multiple autoantibodies (ICA, IAA, GADA, and IA-2A) in adult-onset T1DM compared to childhood-onset cases [14]. In our previous T1DM study (mean age: 15 years, diabetes duration: ≤ 3 months), the prevalence of autoantibodies was as follows: ICA 63%, GADA 75.1%, and IAA 27.1% [30]. In the present study, the prevalence of GADA (65.2% vs. 35%) and ICA (45% vs. 35.6%) was higher in the adult-onset group compared to the child–adolescent-onset group. We believe that the fact that IA-2A, IAA, and ZnT8A are not routinely measured in our laboratory contributes to this situation.

Patients with T1DM are more likely to develop other autoimmunities, the most common of which is autoimmune thyroid disease. Existing literature shows that approximately one-third of individuals with T1DM develop autoimmune thyroid disease within a few years, and this proportion increases up to 50% in TPOA-positive individuals [31–33]. Clinical manifestations of autoimmunity are common in women [33]. In our previous study of adult-onset T1DM, the overall prevalence of autoimmune diseases was 26.2%, with females being more affected than males. Autoimmune thyroid disease was the most common (87%), followed by pernicious anemia, vitiligo, celiac, and premature gonadal failure [34]. The prevalence of TPOA and GPCA in the first 3 months of T1DM onset was 17.8% and 8.2% in our previous study of childhood T1DM [30]. In the present cohort, 43% of child–adolescent-onset and 41.5% of adult-onset patients had at least one autoimmune disease, of which more than 80% were women with autoimmune thyroiditis.

Adults with T1DM were characterized by longer duration of symptoms, higher BMI, and milder metabolic decompensation with lower HbA1c on admission compared to children [14, 35]. In contrast, we did not find any significant difference between child–adolescent-onset and adult-onset T1DM groups with respect to HbA1c, FPG, insulin dose, and lipid profile at onset, as well as the development of hypertension and microvascular and macrovascular complications during follow-up. Longer duration of diabetes was associated with the development of microvascular complications in both groups (*p* < 0.001 and *p* = 0.008). Consistent with others [25, 35], our child–adolescent-onset patients had lower C-peptide levels on admission.

Age-at-onset of diabetes and glycemic control are predictors of microvascular complications. In the VISS study, HbA1c trajectory for 5–8 years after diagnosis strongly predicted severe microangiopathy. In women with childhood-onset diabetes, very high HbA1c during adolescence was associated with a higher prevalence of proliferative retinopathy [36]. A strong positive association was found between long-term mean HbA1c and the occurrence of background and proliferative retinopathy [37, 38]. Younger age-at-onset, CKD, and elevated TG in T1DM may increase the risk of progression to proliferative retinopathy.

Diabetic nephropathy is a critical complication of T1DM. In a cross-sectional study, the prevalence of nephropathy was 16.7%; younger age-at-onset, longer duration of diabetes, HbA1c, diabetic retinopathy, hypertension, elevated TG, and the use of angiotensin-converting enzyme inhibitors/angiotensin receptor blockers (ACEI/ARB) were associated with nephropathy [39]. Data from the NHANES 2015–2018 and an observational study from Ireland showed that the prevalence of CKD in adult T1DM was 21.5% and 18% [40, 41]. In the Swedish National Diabetes Registry, 8.4% of adults 18–49 years and 22.1% of those 50–59 years were reported to have moderate to severe CKD; inadequate glycemic control in the first 5 years of diabetes accelerates the onset of microalbuminuria and nephropathy [42]. In a study comparing German and French cohorts of adult T1DM, the prevalence of retinopathy was 14.4% and 39.1%, nephropathy 20.2% and 16.2%, declined eGFR 5% and 2.8%, increased UACR 15.6% and 13.8%, and neuropathy 48.1% and 27.9% following an average of 21 years [43]. It is noteworthy that we did not find a significant difference between the child–adolescent-onset and adult-onset cohorts in retinopathy (20.6% vs. 15.5%), neuropathy (19% vs. 20%), increased UACR (25% vs. 17.6%), and declined eGFR (5% vs. 5.1%). Consistent with others [39, 42, 43], diabetes duration (*p* = 0.007 and *p* = 0.008) and hypertension (*p* = 0.010and *p* = 0.010) were risk factors of microvascular complications in our child–adolescent-onset and adult-onset T1DM groups. In addition, dyslipidemia/hyperlipidemia (*p* = 0.037) in the child–adolescent-onset group and initial FPG (*p* = 0.014) in the adult-onset group were other predictors of microvascular complications.

Available data suggest that younger-onset T1DM differs from adult-onset T1DM in the development of macrovascular complications. Cardiovascular risk is significantly higher in patients with younger-onset T1DM. Nevertheless, in our cohort, we did not find a significant difference between child–adolescent-onset and adult-onset T1DM in CAD, CVA, PAD, and DFU. In the child–adolescent-onset group, older age (*p* = 0.024) and elevated TG (*p* = 0.013) were associated with increased risk, whereas in the adult-onset group, age-at-onset (*p* = 0.024), dyslipidemia/hyperlipidemia (*p* = 0.03), declined eGFR (*p* = 0.003), and neuropathy (*p* = 0.001) were defined as factors increasing the risk of macrovascular complications. In addition, other risk factors and smoking contribute to cardiovascular disease in adult-onset T1DM. Cumulative tobacco consumption in T1DM patients is associated with atherosclerosis in a dose-dependent manner [44]. In our study, 29% of adult-onset and 10% of child–adolescent-onset cases were smokers. In addition, 62.1% of adult-onset and 26.3% of child–adolescent-onset patients had overweight/obesity.

One of the interesting findings of our study was that hypertension and dyslipidemia in the childhood-onset group and hypertension in the adult-onset group were risk factors for microvascular complications, whereas nephropathy and neuropathy were risk factors for macrovascular complications in the adult-onset group. Diabetic vascular complications share common pathophysiological mechanisms, but the relationship between diabetes-related macrovascular complications and incident diabetic microvascular complications remains unclear. Individual and cumulative macrovascular complications confer an independent risk for incident microvascular complications in patients with T1DM [45]. In our child–adolescent-onset and adult-onset T1DM groups, 10.7% and 18.4% of patients with microangiopathy, respectively, had at least one macroangiopathy, whereas 80% and 78.3% of patients with macroangiopathy, respectively, had at least one microangiopathy.

Endogenous insulin reserve declines more rapidly in childhood T1DM than in adult-onset T1DM. The initial C-peptide level in our child–adolescent-onset group was significantly lower than in the adult-onset group (0.033 vs. 0.54 ng/mL). Delayed presentation of child–adolescent-onset cases to healthcare facilities, often due to lack of parental awareness, may have contributed to this situation. In addition, C-peptide levels were approximately three times lower in child–adolescent-onset patients who developed microvascular complications than in those without complications, although we were unable to establish a definite threshold in this group. We defined a threshold for initial C-peptide levels associated with the later development of microvascular complications in our adult-onset T1DM group only. C-peptide levels below 0.41 ng/mL were associated with the development of microvascular complications with reasonable sensitivity (65.5%) and specificity (64.9%). Likewise, in this group, we identified baseline HbA1c 8.55% as a threshold that predicted the development of microvascular complications with similar sensitivity (65.5%) but lower specificity (53.8%).

Another interesting finding of our study is metformin use in adult T1DM. Although metformin as an adjunct to insulin in T1DM does not provide a significant improvement in glycemic control, it is effective in maintaining weight and reducing insulin doses [46, 47]. Since more than half of our adult patients were overweight/obese and a third had dyslipidemia, almost a quarter of our adult patients were on metformin. Unfortunately, despite the high rates of comorbidities, especially in the adult-onset T1DM group, preventive therapies such as ASA, statins, and ACEI/ARB were used less frequently.

Our study has several limitations. First, the retrospective study design is an important limitation. Second, cases diagnosed with T1DM before the age of 18 years were initially followed by pediatric endocrine units and later transferred to us. Therefore, the clinical and laboratory data of these cases were obtained from medical reports and EHRs. This may have resulted in some loss of data, especially autoantibodies and C-peptide measured at the time of diagnosis. Because our study was based on a clinical diagnosis of T1DM, the third limitation may be that we excluded cases of latent autoimmune diabetes in adults (LADA). Finally, since our study is based on data from a tertiary care institution, selection bias may be a potential issue. Also, the results depended on a single-center experience; therefore, they cannot be generalized, and they should be interpreted with caution. However, the large size of the study cohort, the fact that we had child–adolescent and adult-onset groups followed by the same diabetes team for a period of the median of 5 years, and routine screening for microvascular and macrovascular complications performed as per guidelines [6] are the main strengths of our study.

## 5. Conclusion

Technological advances in monitoring and treatment over the past few decades have enabled people with T1DM, whether in childhood or adulthood, to survive for longer years. The results of our study showed that contrary to what was previously known, adult-onset T1DM patients can develop microvascular and macrovascular complications at rates similar to those of childhood-onset with adequate follow-up. In adult-onset T1DM, lower C-peptide reserve and poorer glycemic control at baseline may predict the future development of microvascular complications. In addition, cardiovascular factors such as hypertension and dyslipidemia may increase the risk of microvascular complications, while neuropathy and nephropathy may increase the risk of macrovascular complications in patients with T1DM. Regular screening for long-term complications, monitoring of cardiovascular risk factors, and timely initiation of preventive therapies are of paramount importance in all patients with T1DM, especially in adult-onset cases.

## Figures and Tables

**Table 1:** Demographic, clinical, and laboratory features of the study groups at baseline.

**Variables**	**Total group (** **n** = 490**)**	**a. Child–adolescent-onset T1DM (** **n** = 200**)**	**b. Adult-onset T1DM (** **n** = 290**)**	**p** ** value (a vs. b)**
Age, years, mean ± SD	38.1 ± 12.6	31 ± 7.5	43 ± 13	**< 0.001**
Gender, female, *n* (%)	266 (54.3)	109 (54.5)	157 (54)	0.900
Diabetes duration (years), median (IQR)	16 (12)	20 (12)	14 (12)	**< 0.001**
Follow-up duration (years), median (IQR)	5 (5)	5 (5)	6 (6)	0.100
Family history, *n* (%)	291/470 (62)	116/197 (59)	175/273 (64)	0.250
T1DM	87/470 (18.5)	39/197 (19.8)	48/273 (17.6)	0.542
T2DM	226/470 (48.1)	87/197 (44.2)	139/273 (51)	0.148
BMI (kg/m^2^), mean ± SD	24.2 ± 4.4	22.6 ± 3.2	25 ± 4.6	**< 0.001**
Smoking, *n* (%)	100/432 (23.1)	29/186 (15.6)	71/246 (29.0)	**0.001**
Alcohol, *n* (%)	49/465 (10.5)	19/192 (10.0)	30/273 (11)	0.700
Presentation with DKA, *n* (%)	93/407 (22.9)	56/163 (34.4)	37/244 (15.2)	**< 0.001**
≥ 1 islet autoantibody, *n* (%)	128/177 (72.3)	26/45 (57.8)	102/132 (77.3)	**0.012**
GADA, *n* (%)	98/178 (55.1)	15/43 (34.9)	88/135 (65.2)	**< 0.001**
ICA, *n* (%)	72/170 (42.4)	17/41 (41.5)	56/119 (47.0)	0.300
FPG at presentation (mg/dL), median (IQR)	228.4 (115.0)	231.6 (107.0)	227.6 (120.0)	0.700
HbA1c at presentation (%), median (IQR)	8.8 (2.3)	8.7 (2.1)	8.95 (2.4)	0.200
Insulin dose at baseline (IU/kg/day), median (IQR)	0.32 (0.2)	0.36 (0.22)	0.30 (0.2)	0.074
C-peptide (ng/mL), median (IQR)	0.35 (1)	0.033 (0.4)	0.54 (1.2)	**< 0.001**
eGFR (mL/min/1.73 m^2^), median (IQR)	122 (28)	127 (19)	116 (30)	**0.001**
Total-C (mg/dL), median (IQR)	175.6 (38.8)	173.7 (41.0)	177.3 (37.0)	0.400
LDL-C (mg/dL), median (IQR)	103.2 (33.9)	103.0 (36.0)	103.5 (33.0)	0.900
HDL-C (mg/dL), median (IQR)	53.82 (15.9)	55.5 (14.0)	52.7 (17.0)	0.100
TG (mg/dL), median (IQR)	102.7 (68.6)	97.0 (72.0)	106.7 (67.0)	0.200

*Note:* Bold values are statistically significant.

Abbreviations: BMI, body mass index; DKA, diabetic ketoacidosis; eGFR, estimated glomerular filtration rate; FPG, fasting plasma glucose; GADA, glutamic acid decarboxylase autoantibody; HbA1c, glycated hemoglobin A1c; HDL-C, high-density lipoprotein cholesterol; ICA, islet cell cytoplasmic autoantibody; IQR, interquartile range; LDL-C, low-density lipoprotein cholesterol; SD, standard deviation; T1DM, Type 1 diabetes mellitus; T2DM, Type 2 diabetes mellitus; TG, triglycerides; Total-C, total cholesterol.

**Table 2:** Development of micro- and macrovascular complications and comorbidities during follow-up.

**Variables**	**Total group (** **n** = 490**)**	**a. Child–adolescent-onset T1DM (** **n** = 200**)**	**b. Adult-onset T1DM (** **n** = 290**)**	**p** ** value (a vs. b)**
Any microvascular complication, *n* (%)	171/485 (35.3)	74/200 (37.0)	97/285 (34.0)	0.500
Retinopathy, *n* (%)	84/476 (17.6)	41/199 (20.6)	43/277 (15.5)	0.150
Neuropathy, *n* (%)	93/475 (19.6)	38/200 (19.0)	55/275 (20.0)	0.800
UACR > 30 mg/g, *n* (%)	97/468 (20.7)	49/196 (25)	48/272 (17.6)	0.053
eGFR < 60 mL/min/1.73 m^2^, *n* (%)	22/436 (5.0)	9/181 (5)	13/255 (5.1)	1.000
Any macrovascular complication, *n* (%)	33/486 (6.8)	10/200 (5.0)	23/286 (8.0)	0.200
CAD, *n* (%)	19/483 (3.9)	4/200 (2.0)	15/283 (5.3)	0.070
CVA, *n* (%)	4/486 (0.8)	2/200 (1.0)	2/286 (0.7)	0.700
PAD, *n* (%)	5/467 (1.1)	1/200 (0.5)	4/267 (1.5)	0.300
DFU, *n* (%)	11/486 (2.3)	5/200 (2.5)	6/286 (2.1)	0.800
Hypertension, *n* (%)	61/302 (20.2)	3/16 (18.5)	58/286 (20.3)	0.600
Dyslipidemia/hyperlipidemia, *n* (%)	133/482 (27.6)	40/200 (20.0)	93/282 (33.0)	**0.002**
Overweight, *n* (%)	85/228 (37.3)	19/79 (24.0)	66/149 (44.3)	**0.003**
Obesity, *n* (%)	14/228 (14.0)	2/86 (2.3)	30/169 (17.8)	**< 0.001**
Other autoimmune diseases, *n* (%)	176/416 (42.1)	78/180 (43.0)	98/236 (41.5)	0.800
Hashimoto's thyroiditis	148/416 (35.6)	63/181 (34.8)	85/236 (35.7)	0.360
Graves' disease	14 (3.3)	5/181 (2.8)	9 (3.8)	0.132
Celiac disease	7 (1.7)	5/181 (2.8)	2 (0.8)	0.131
Atrophic gastritis	13 (3.1)	4/181 (2.2)	1/236 (0.4)	0.097
Rheumatoid arthritis	2 (0.5)	1 (0.6)	1 (0.4)	0.554
Medications, *n* (%)				
Metformin	74/478 (15.5)	8/198 (4)	66/280 (23.6)	**< 0.001**
ASA	51 (10.5)	12 (6)	39 (13.5)	**0.007**
Statin	80/450 (17.8)	22/189 (11.6)	58/261 (22.2)	**0.004**
Overall antihypertensive drugs	100 (20.4)	36 (18)	64 (22)	0.795
ACEI/ARB	61 (12.5)	21 (10)	40 (13.8)	0.604
L-thyroxine	76 (15.5)	30 (15)	46 (15.9)	0.795

*Note:* Bold values are statistically significant.

Abbreviations: ACEI/ARB, angiotensin-converting enzyme inhibitor or angiotensin receptor blocker; ASA, acetylsalicylic acid; CAD, coronary artery disease; CVA, cerebrovascular accident; DFU, diabetic foot ulcer; eGFR, estimated glomerular filtration rate; PAD, peripheral artery disease; T1DM, Type 1 diabetes mellitus; UACR, urine albumin-to-creatinine ratio.

**Table 3:** Univariate and multivariate analyses of associated factors with microvascular complications in patients with Type 1 diabetes mellitus.

**Variables**	**Child–adolescent-onset T1DM (** **n** = 200**)**				**Adult-onset T1DM (** **n** = 290**)**			
	**Univariate analysis**			**Multivariate analysis**	**Univariate analysis**			**Multivariate analysis**
	**No (** **n** = 126**)**	**Yes (** **n** = 74**)**	**p** ** value**	**OR; 95% CI; ** **p**	**No (** **n** = 191**)**	**Yes (** **n** = 98**)**	**p** ** value**	**OR; 95% CI; ** **p** ** value**
Age (years), mean ± SD	28.8 (5.7)	34.9 (8.7)	**< 0.001**	NS	40 (12)	48.5 (13.2)	**< 0.001**	NS
Gender, female, *n* (%)	68 (54)	42 (56)	0.800		104 (54.5)	55 (56.0)	0.800	
Age-at-onset (years), median (IQR)	11.3 (4.6)	10.3 (4.0)	0.150	NS	27.2 (9.2)	29.3 (9.0)	0.060	NS
Duration of diabetes (years), median (IQR)	17.5 (7.0)	24.8 (8.8)	**< 0.001**	**1.2; 1.05–1.37; 0.007**	12.9 (6.9)	19.5 (10.5)	**< 0.001**	**1.08; 1.02–1.13; 0.008**
Follow-up duration (years), median (IQR)	6.7 (4.8)	6 (3.8)	0.300		6.5 (4.3)	8.1 (4.9)	**0.006**	NS
Smoking, *n* (%)	16/120 (13.3)	14/67 (21)	0.200	NS	44/166 (26.5)	28/82 (34)	0.200	NS
DKA, *n* (%)	34/104 (32.7)	22/60 (36.7)	0.600		26/166 (15.7)	11/79 (14)	0.700	
GADA, *n* (%)	10/27 (37)	5/16 (31.3)	0.700		60/91 (66)	28/4 (63.6)	0.800	
ICA, *n* (%)	12/30 (40)	5/16 (31.3)	0.600		39/84 (46.4)	17/41 (41.5)	0.600	
BMI (kg/m^2^), median (IQR)	22.7 (3.2)	22.6 (3.4)	1.000		24.6 (4.8)	25.7 (4.3)	0.100	NS
FPG (mg/dL), median (IQR)	237 (104)	223 (111)	0.400		214 (108)	253 (138)	**0.01**	**1.005; 1.001–1.009; 0.024**
HbA1c (%), median (IQR)	8.7 (2.2)	8.6 (1.9)	0.800		8.7 (2.5)	9.5 (2.1)	**0.009**	NS
IDA-HbA1c > 8.9%, *n* (%)	45/116 (38.8)	22/70 (31.4)	0.300		67/169 (39.6)	45/87 (51.7)	0.065	NS
Total-C (mg/dL), median (IQR)	168 (39)	183 (42)	**0.035**	NS	173 (34)	184 ± 42	0.070	NS
LDL-C (mg/dL), median (IQR)	97.3 (31)	112 (41)	**0.006**	NS	101 (33)	108 (32)	0.100	NS
HDL-C (mg/dL), median (IQR)	54 (13.3)	57.6 (14.6)	0.100	NS	52.6 (17)	52.9 (17.5)	0.900	
TG (mg/dL), median (IQR)	88.7 (54.4)	110 (91)	0.060	NS	97.6 (59.3)	124 (76)	**0.030**	NS
Hypertension, *n* (%)	5 (4)	32 (43)	**< 0.001**	**18.6; 1.9–184.6; 0.010**	18 (9.4)	40 (41)	**< 0.001**	**3.9; 1.4–10.8; 0.010**
Dyslipidemia/hyperlipidemia, *n* (%)	16 (12.7)	224 (32)	**0.001**	**7.1; 1.2–45.2; 0.037**	47 (25)	46 (47)	**< 0.001**	
Overweight, *n* (%)	12/53 (22.6)	7/26 (27)	0.700		39/96 (40.6)	27/53 (51)	0.220	
Obesity, *n* (%)	1/58 (1.7)	1/28 (3.6)	0.600		15/110 (13.6)	15/59 (25.4)	0.056	NS
Any macrovascular comp, *n* (%)	2 (1.6)	8 (10.7)	**0.004**	NS	5 (2.6)	18 (18.4)	**< 0.001**	NS
CAD, *n* (%)	1 (0.8)	3 (4.1)	0.100	NS	4 (2.1)	11 (11.2)	**0.001**	NS
CVA, *n* (%)	0	2 (2.7)	0.065	NS	1 (0.5)	1 (1)	0.6	
PAD, *n* (%)	0	1 (1.3)	0.200	NS	0	4 (4.1)	**0.005**	NS
DFU, *n* (%)	1 (0.8)	4 (5.3)	**0.046**	NS	0	6 (6)	**0.001**	NS
Other autoimmune diseases, *n* (%)	48/112 (43)	30/69 (43.5)	0.900		59/156 (37.8)	39/82 (47.6)	0.150	NS

*Note:* Bold values are statistically significant.

Abbreviations: BMI, body mass index; CAD, coronary artery disease; CI, confidence intervals; CVA, cerebrovascular accident; DFU, diabetic foot ulcer; DKA, diabetic ketoacidosis; FPG, fasting blood glucose; GADA, glutamic acid decarboxylase autoantibody; HbA1c, glycated hemoglobin A1c; HDL-C, high-density lipoprotein cholesterol; ICA, islet cell cytoplasmic autoantibodies; IDA-HbA1c, insulin dose-adjusted HbA1c; IQR, interquartile range; LDL-C, low-density lipoprotein cholesterol; NS, nonsignificant; OR, odds ratio; PAD, peripheral artery disease; SD, standard deviation; T1DM, Type 1 diabetes mellitus; TG, triglycerides; Total-C, total cholesterol.

**Table 4:** Univariate and multivariate analyses of associated factors with macrovascular complications in patients with Type 1 diabetes mellitus.

**Variables**	**Child–adolescent-onset T1DM (** **n** = 200**)**				**Adult-onset T1DM (** **n** = 290**)**			
	**Univariate analysis**			**Multivariate analysis**	**Univariate analysis**			**Multivariate analysis**
	**No (** **n** = 190**)**	**Yes (** **n** = 10**)**	**p** ** value**	**OR; 95% CI; ** **p**	**No (** **n** = 267**)**	**Yes (** **n** = 23**)**	**p** ** value**	**OR; 95% CI; ** **p** ** value**
Age (years), median (IQR)	30.5 (7.0)	41.9 (9.5)	**< 0.001**	**1.2; 1.05–1.26; 0.002**	41.9 (12.6)	54.8 (13.7)	**< 0.001**	NS
Gender, female, *n* (%)	106 (55.5)	4 (40.0)	0.300		148 (55.6)	11 (47.8)	0.500	
Age-at-onset (years), median (IQR)	10.9 (4.5)	10.4 (2.9)	0.700		27.4 (8.4)	34.2 (14.2)	**0.001**	**1.07; 1.01–1.13; 0.024**
Duration of diabetes (years), median (IQR)	19.6 (7.9)	31.5 (10.5)	**< 0.001**	NS	14.6 (8.6)	21.3 (10.0)	**< 0.001**	NS
Follow-up duration (years), median (IQR)	6.45 (4.5)	5.4 (2.9)	0.500		7.0 (4.6)	8.4 (4.9)	0.200	
Smoking, *n* (%)	27/179 (15)	3/8 (37.5)	0.09	NS	67/232 (29)	5/16 (31.3)	0.800	
DKA, *n* (%)	55/158 (35.0)	1/6 (16.7)	0.400		37/231 (16.0)	0	0.100	
GADA, *n* (%)	15/42 (35.7)	0/1	0.500		79/124 (63.7)	9/11 (81.8)	0.200	
ICA, *n* (%)	17/45 (38.0)	0/1	0.400		47/114 (41.2)	9/11 (81.8)	**0.010**	NS
BMI (kg/m^2^), median (IQR)	22.7 (3.2)	21.4 (3.0)	0.500		24.9 (4.8)	25.6 (2.8)	0.600	
FPG (mg/dL)	233 (109)	207 (65)	0.400		224 (119)	270 (132)	0.0900	NS
HbA1c (%), median (IQR)	8.7 (2.2)	8.3 (1.6)	0.600		8.9 (2.5)	9.2 (1.9)	0.600	
C-peptide (ng/mL), median (IQR)	0.036 (0.4)	0.016 (0.1)	0.400		0.58 (1.2)	0.14 (1)	0.200	
Total-C (mg/dL), median (IQR)	172 (41)	197 (29)	0.090	NS	176 (36)	190 (132)	0.200	
LDL-C (mg/dL), median (IQR)	102 (36)	126 (28)	**0.030**	NS	103 (33)	111 (34)	0.300	
HDL-C (mg/dL), median (IQR)	55.6 (13)	51.9 (23)	0.400		52.3 (17.4)	57.6 (14.7)	0.200	
TG (mg/dL), median (IQR)	93.5 (64)	148 (142)	**0.020**	**1.01; 1.002–1.017; 0.013**	106 (66)	113 (72)	0.700	—
Overweight, *n* (%)	19/77 (24.7)	0	0.400		61/139 (44)	5/10 (50)	0.700	
Obesity, *n* (%)	2/83 (2.4)	0	0.800		28/154 (18.2)	2/15 (13.3)	0.600	
Hypertension, *n* (%)	30 (15.5)	7 (70.0)	**< 0.001**	NS	44 (16.5)	64 (61.0)	**< 0.001**	NS
Dyslipidemia/hyperlipidemia, *n* (%)	34 (18.0)	6 (60.0)	**0.001**	NS	76 (28.5)	17 (74.0)	**< 0.001**	**5.7; 1.16–27.9; 0.03**
Any microvascular comp, *n* (%)	67 (35)	8 (80)	**0.004**	NS	80 (30)	18 (78.3)	**< 0.001**	NS
UACR > 30 mg/g, *n* (%)	47 (25)	3/9 (33.3)	0.600		41/251 (16.3)	8 (34.8)	**0.030**	NS
eGFR <60 mL/min, *n* (%)	8/172 (4.7)	1 (10.0)	0.400		8/237 (3.4)	5/19 (26.3)	**< 0.001**	**15.4; 2.6–92.2; 0.003**
Retinopathy, *n* (%)	36 (19)	5 (50)	**0.020**	NS	31/257 (12)	12 (52.2)	**< 0.001**	NS
Neuropathy, *n* (%)	31 (16.3)	7 (70)	**< 0.001**	NS	39/257 (15.2)	16 (69.6)	**< 0.001**	**13.2; 2.8–62.4; 0.001**
Other autoimmune diseases, *n* (%)	76/172 (44.2)	2/9 (22.2)	0.200		90/219 (41.0)	8/19 (42.0)	1.000	

*Note:* Bold values are statistically significant.

Abbreviations: BMI, body mass index; CI, confidence intervals; DKA, diabetic ketoacidosis; FPG, fasting blood glucose; GADA, glutamic acid decarboxylase autoantibody; HbA1c, glycated hemoglobin A1c; HDL-C, high-density lipoprotein cholesterol; ICA, islet cell cytoplasmic autoantibodies; IQR, interquartile range; LDL-C, low-density lipoprotein cholesterol; NS, nonsignificant; OR, odds ratio; T1DM, Type 1 diabetes mellitus; TG, triglycerides; Total-C, total cholesterol.

**Table 5:** Comparison of child–adolescent-onset and adult-onset T1DM groups after controlled for confoundings: (a) Model-1 (unadjusted), (b) Model-2 (adjusted for age and diabetes duration), and (c) Model-3 (adjusted for age, diabetes duration, smoking, and alcohol consumption).

**(a) Model-1 (unadjusted)**					
**Variables**	**Total group (** **n** = 490**)**	**a. Child–adolescent-onset T1DM (** **n** = 200**)**	**b. Adult-onset T1DM (** **n** = 290**)**	**p** ** value (a vs. b)**	
Any microvascular complication, *n* (%)	171/485 (35.3)	74/200 (37.0)	97/285 (34.0)	0.50	
Retinopathy	84/476 (17.6)	41/199 (20.6)	43/277 (15.5)	0.15	
Neuropathy	93/475 (19.6)	38/200 (19.0)	55/275 (20.0)	0.80	
UACR > 30 mg/g	97/468 (20.7)	49/196 (25)	48/272 (17.6)	0.053	
eGFR < 60 mL/min/1.73 m^2^	22/436 (5.0)	9/181 (5)	13/255 (5.1)	1.00	
Any macrovascular complication, *n* (%)	33/486 (6.8)	10/200 (5.0)	23/286 (8.0)	0.20	
CAD	19/483 (3.9)	4/200 (2.0)	15/283 (5.3)	0.07	
CVA	4/486 (0.8)	2/200 (1.0)	2/286 (0.7)	0.70	
PAD	5/467 (1.1)	1/200 (0.5)	4/267 (1.5)	0.30	
DFU	11/486 (2.3)	5/200 (2.5)	6/286 (2.1)	0.80	
Comorbidities, *n* (%)					
Hypertension	61/302 (20.2)	3/16 (18.5)	58/286 (20.3)	0.60	
Dyslipidemia/hyperlipidemia	133/482 (27.6)	40/200 (20.0)	93/282 (33.0)	**0.002**	
Overweight	85/228 (37.3)	19/79 (24.0)	66/149 (44.3)	**0.003**	
Obesity	14/228 (14.0)	2/86 (2.3)	30/169 (17.8)	**< 0.001**	
Other autoimmune diseases, *n* (%)	176/416 (42.1)	78/180 (43.0)	98/236 (41.5)	0.80	
Hashimoto's thyroiditis	148/416 (35.6)	63/181 (34.8)	85/236 (35.7)	0.36	
Graves' disease	14 (3.3)	5/181 (2.8)	9 (3.8)	0.132	
Celiac disease	7 (1.7)	5/181 (2.8)	2 (0.8)	0.131	
Atrophic gastritis	13 (3.1)	4/181 (2.2)	1/236 (0.4)	0.097	
Rheumatoid arthritis	2 (0.5)	1 (0.6)	1 (0.4)	0.554	
Concomitant medications, *n* (%)					
Metformin	74/478 (15.5)	8/198 (4)	66/280 (23.6)	**< 0.001**	
ASA	51 (10.5)	12 (6)	39 (13.5)	**0.007**	
Statin	80/450 (17.8)	22/189 (11.6)	58/261 (22.2)	**0.004**	
Antihypertensive drugs	100 (20.4)	36 (18)	64 (22)	0.795	
ACEI/ARB	61 (12.5)	21 (10)	40 (13.8)	0.604	
L-thyroxine	76 (15.5)	30 (15)	46 (15.9)	0.795	

**(b) Model-2 (adjusted for age and diabetes duration)**					
**Variables**	**Total group (** **n** = 490**)**	**a. Child–adolescent-onset (** **n** = 200**)**	**b. Adult-onset ( ** **n** = 290**)**	**p** ** value (a vs. b)**	**Adjusted ** **p** ** value**
Any microvascular complication, *n* (%)	171/485 (35.3)	74/200 (37.0)	97/285 (34.0)	0.50	0.249
Retinopathy, *n* (%)	84/476 (17.6)	41/199 (20.6)	43/277 (15.5)	0.15	0.501
Neuropathy, *n* (%)	93/475 (19.6)	38/200 (19.0)	55/275 (20.0)	0.80	0.434
UACR > 30 mg/g, *n* (%)	97/468 (20.7)	49/196 (25)	48/272 (17.6)	0.053	0.280
eGFR < 60 mL/min/1.73 m^2^, *n* (%)	22/436 (5.0)	9/181 (5)	13/255 (5.1)	1.00	0.063
Any macrovascular complication, *n* (%)	33/486 (6.8)	10/200 (5.0)	23/286 (8.0)	0.20	**0.007**
CAD	19/483 (3.9)	4/200 (2.0)	15/283 (5.3)	0.07	**< 0.001**
CVA	4/486 (0.8)	2/200 (1.0)	2/286 (0.7)	0.70	0.975
PAD	5/467 (1.1)	1/200 (0.5)	4/267 (1.5)	0.30	0.912
DFU	11/486 (2.3)	5/200 (2.5)	6/286 (2.1)	0.80	0.470

**(c) Model-3 (adjusted for age, diabetes duration, smoking, and alcohol consumption)**					
**Variables**	**Total group (** **n** = 490**)**	**a. Child–adolescent-onset (** **n** = 200**)**	**b. Adult-onset ( ** **n** = 290**)**	**p** ** value (a vs. b)**	**Adjusted ** **p** ** value**
Any microvascular complication, *n* (%)	171/485 (35.3)	74/200 (37.0)	97/285 (34.0)	0.50	0.249
Retinopathy	84/476 (17.6)	41/199 (20.6)	43/277 (15.5)	0.15	0.501
Neuropathy	93/475 (19.6)	38/200 (19.0)	55/275 (20.0)	0.80	0.434
UACR > 30 mg/g	97/468 (20.7)	49/196 (25)	48/272 (17.6)	0.053	0.280
eGFR < 60 mL/min/1.73 m^2^	22/436 (5.0)	9/181 (5)	13/255 (5.1)	1.00	0.063
Any macrovascular complication, *n* (%)	33/486 (6.8)	10/200 (5.0)	23/286 (8.0)	0.20	**0.007**
CAD	19/483 (3.9)	4/200 (2.0)	15/283 (5.3)	0.07	**< 0.001**
CVA	4/486 (0.8)	2/200 (1.0)	2/286 (0.7)	0.70	0.975
PAD	5/467 (1.1)	1/200 (0.5)	4/267 (1.5)	0.30	0.912
DFU	11/486 (2.3)	5/200 (2.5)	6/286 (2.1)	0.80	0.470

*Note:* Bold values are statistically significant.

Abbreviations: ACEI, angiotensin-converting enzyme inhibitors; ARB, angiotensin receptor blockers; ASA, acetylsalicylic acid; CAD, coronary artery disease; CVA, cerebrovascular accident; DFU, diabetic foot ulcers; eGFR, estimated glomerular filtration rate; PAD, peripheral artery disease; T1DM, Type 1 diabetes mellitus; UACR, urine albumin-to-creatinine ratio.

## Data Availability

Study datasets are available from the corresponding author upon reasonable request.

## Supporting Information

Supporting Information Additional supporting information can be found online in the Supporting Information section. Table S1: Comparison of clinical and laboratory findings in Type 1 diabetes patients with and without autoantibody results. Table S2: Comparison of clinical and laboratory findings in patients with child–adolescent-onset and adult-onset Type 1 diabetes mellitus according to the autoantibody availability. Table S3: Comparison of clinical and laboratory findings according to age-at-onset status in patients who developed microvascular complications. Table S4: Comparison of clinical and laboratory findings according to age at onset status in patients who developed macrovascular complications.
