# Supplementary material for: Clinical Characteristics and Development of Complications Differ Between Adult-Onset and Child–Adolescent-Onset Type 1 Diabetes: A Report From a Tertiary Medical Center in Türkiye
Source: J Diabetes Res. 2025 Apr 9;2025:8860118. doi: 10.1155/jdr/8860118 (PMC12003040; doi:10.1155/jdr/8860118)
Supplement: Supporting Information — Additional supporting information can be found online in the Supporting Information section. Table S1: Comparison of clinical and laboratory findings in Type 1 diabetes patients with and without autoantibody results. Table S2: Comparison of clinical and laboratory findings in patients with child–adolescent-onset and adult-onset Type 1 diabetes mellitus according to the autoantibody availability. Table S3: Comparison of clinical and laboratory findings according to age-at-onset status in patients who developed microvascular complications. Table S4: Comparison of clinical and laboratory findings according to age at onset status in patients who developed macrovascular complications. [file 8860118.f1.docx]

**Suppl. Table 1.** Comparison of clinical and laboratory findings in type 1 diabetes patients with and without autoantibody results

| Variables | Patients with autoantibody results | | | Patients without autoantibody results | | |
| --- | --- | --- | --- | --- | --- | --- |
|  | **Child-adolescent-onset (n=50)** | **Adult-onset**  **(n=140)** | **p value** | **Child-adolescent-onset (n=151)** | **Adult-onset (n=149)** | **p value** |
| Age, years, mean±SD | 27.9±5.2 | 40.6±13.2 | ***<0.001*** | 32±7.9 | 45.3±12.7 | ***<0.001*** |
| Gender, female, n (%) | 22 (44) | 76 (54) | 0.212 | 88 (48.3) | 83 (56) | 0.653 |
| Diabetes duration, years, median (IQR) (range) | 16 (11) (1-37) | 10 (10) (1-39) | ***<0.001*** | 21 (12) (6-47) | 16 (13) (1-45) | ***0.001*** |
| Follow-up duration, years, median (IQR) | 5 (4) | 6 (6) | 0.165 | 5 (5) | 7 (7) | 0.167 |
| Family history, n (%) | 32/49 (65) | 85/135 (63) | 0.770 | 84/148 (57) | 90/138 (65) | 0.143 |
| T1DM | 10 (20) | 17/135 (13) | 0.185 | 29/148 (19.6) | 31/138 (22.5) | 0.552 |
| T2DM | 25 (50) | 75/135 (56) | 0.585 | 62/148 (42) | 64/138 (46.4) | 0.445 |
| BMI, kg/m^2^, mean±SD | 22.4±3.7 | 25±5 | ***0.023*** | 22.7±3 | 24.9±5.7 | ***0.001*** |
| Smoking, n (%) | 6/46 (13) | 33/122 (27) | 0.055 | 24/141 (17) | 39/126 (31) | ***0.007*** |
| Alcohol, n (%) | 5/47 (10.6) | 10/135 (7.4) | 0.488 | 14/146 (9.6) | 20/140 (14.3) | 0.220 |
| Presentation with DKA, n (%) | 15/43 (35) | 17/125 (14) | ***0.002*** | 41/121 (34) | 20/120 (16.7) | ***0.002*** |
| ≥1 islet autoantibody (+), n (%) | 26/45 (58) | 102/132 (77) | ***0.012*** | NA | NA |  |
| GADA, n (%) | 15/43 (35) | 88/135 (65) | ***<0.001*** | NA | NA |  |
| ICA, n (%) | 17/41 (41.5) | 56/119 (47) | 0.535 | NA | NA |  |
| FPG at presentation, mg/dL, median (IQR) | 240 (189) | 210 (171) | 0.473 | 220 (139) | 212 (142) | 0.472 |
| HbA1c at presentation, %, median (IQR) | 9.1 (2.6) | 9.2 (2.6) | 0.521 | 8.4 (2.2) | 8.5 (3.1) | 0.325 |
| Insulin dose at baseline, IU/kg/day, median (IQR) | 0.33 (0.3) | 0.3 (0.2) | 0.289 | 0.37 (0.2) | 0.31 (0.2) | 0.364 |
| C-peptide, ng/mL, median (IQR) | 0.36 (0.9) | 0.77 (1.2) | ***0.003*** | 0.02 (0.1) | 0.2 (0.7) | ***0.014*** |
| eGFR, mL/min/1.73 m^2^, median (IQR) | 129 (11) | 117 (25) | ***0.005*** | 125 (21) | 115 (39) | ***0.022*** |
| Total-C, mg/dL, median (IQR) | 160 (43) | 168 (46) | 0.076 | 168 (50) | 172 (48) | 0.539 |
| LDL-C, mg/dL, median (IQR) | 98.5 (38) | 98.5 (37) | 0.283 | 100 (44) | 100 (44) | 0.856 |
| HDL-C, mg/dL, median (IQR) | 50.5 (19) | 50.5 (21) | 0.620 | 55 (17) | 49 (22) | ***0.017*** |
| TG, mg/dL, median (IQR) | 78 (61) | 79 (56) | 0.618 | 74 (46) | 98 (80) | ***0.002*** |
| Follow-up | | | | | | |
| Other autoimmune diseases, n (%) | 21/47 (45) | 48/121 (40) | 0.553 | 57/134 (42.5) | 50/117 (42.7) | 0.975 |
| Hypertension, n (%) | 6 (12) | 25 (18) | 0.336 | 31 (20.5) | 33 (22) | 0.732 |
| Dyslipidemia/hyperlipidemia, n (%) | 7 (14) | 41 (29) | ***0.033*** | 33 (22) | 52 (35) | ***0.010*** |
| Overweight, n (%) | 4/21 (19) | 27/60 (45) | ***0.035*** | 15/58 (26) | 39/89 (44) | ***0.027*** |
| Obesity, n (%) | 1/22 (4.5) | 13/72 (18) | 0.119 | 1/64 (1.6) | 17/97 (17.5) | ***0.002*** |
| Any macrovascular complications, n (%) | 1 (2) | 12 (8.6) | 0.114 | 9 (6) | 11 (7.4) | 0.621 |
| CAD, n (%) | 0 | 8 (5.7) | 0.084 | 4 (2.7) | 7 (4.7) | 0.345 |
| CVA, n (%) | 0 | 1 (0.7) | 0.549 | 2 (1.3) | 1 (0.7) | 0.574 |
| PAD, n (%) | 0 | 2 (1.4) | 0.394 | 1 (0.7) | 2 (1.4) | 0.550 |
| DFU, n (%) | 1 (2) | 2 (1.4) | 0.781 | 4 (2.6) | 4 (2.7) | 0.977 |
| Any microvascular complications, n (%) | 19 (38) | 45 (32) | 0.452 | 56 (37) | 53 (35.6) | 0.785 |
| Retinopathy, n (%) | 5 (10) | 14 (10) | 1.000 | 36 (24) | 29 (21) | 0.503 |
| Neuropathy, n (%) | 12 (24) | 26 (19) | 0.423 | 26 (17.3) | 29 (20.6) | 0.481 |
| UACR >30 mg/g, n (%) | 13 (26) | 27 (20) | 0.353 | 37 (25) | 22 (16) | 0.059 |
| eGFR <60 mL/min/1.73 m^2^, n (%) | 0 | 3 (2.3) | 0.284 | 9/133 (6.8) | 10/126 (8) | 0.718 |

SD, standard deviation; IQR, interquartile range; T1DM, type 1 diabetes mellitus; T2DM, type 2 diabetes mellitus; BMI, body mass index; DKA, diabetic ketoacidosis; GADA, glutamic acid decarboxylase autoantibody; ICA, islet cell cytoplasmic autoantibody; FPG, fasting plasma glucose; HbA1c, glycated hemoglobin A1c; eGFR, estimated glomerular filtration rate; Total-C, total cholesterol; LDL-C, low-density lipoprotein cholesterol; HDL-C, high-density lipoprotein cholesterol; TG, triglycerides; CAD, coronary artery disease; CVA, cerebrovascular accident; PAD, peripheral artery disease; DFU, diabetic foot ulcer; UACR, urine albumin-to-creatinine ratio.

**Suppl. Table 2.** Comparison of clinical and laboratory findings in patients with child-adolescent-onset and adult-onset type 1 diabetes mellitus according to the autoantibody availability

| Variables | Child-adolescent-onset T1DM | | | Adult-onset T1DM | | |
| --- | --- | --- | --- | --- | --- | --- |
|  | **Autoantibody non-available (n=151)** | **Autoantibody available (n=50)** | **p value** | **Autoantibody non-available (n=149)** | **Autoantibody available (n=140)** | **p value** |
| Age, years, mean±SD | 32±7.9 | 27.9±5.2 | ***<0.001*** | 45.3±12.7 | 40.6±13.2 | ***0.002*** |
| Gender, female, n (%) | 88 (58.3) | 22 (44) | 0.079 | 83 (55.7) | 76 (54.3) | 0.809 |
| DM duration, years, median (IQR), (range) | 21 (12) (6-47) | 16 (11) (1-37) | ***<0.001*** | 16 (13) (1-45) | 10 (10) (1-39) | ***<0.001*** |
| Follow-up duration, years, median (IQR) | 5 (5) | 5 (4) | 0.271 | 7 (7) | 6 (6) | *0.353* |
| Family history, n (%) | 84/148 (56.8) | 32 (64) | 0.292 | 90/138 (65.2) | 85/135 (63) | 0.698 |
| T1DM | 29/148 (19.6) | 10 (20) | 0.901 | 31/138 (22.5) | 17/135 (12.6) | ***0.032*** |
| T2DM | 62/148 (42) | 25 (50) | 0.265 | 64/138 (46.4) | 75/135 (55.6) | 0.129 |
| BMI, kg/m^2^, mean±SD | 22.7±3 | 22.4±3.7 | 0.665 | 24.9±4.4 | 25±5 | 0.906 |
| Smoking, n (%) | 24/141 (17) | 6/46 (13) | 0.523 | 39/126 (31) | 33/122 (27) | 0.498 |
| Alcohol, n (%) | 14/146 (9.6) | 5/47 (10.6) | 0.834 | 20/140 (14.3) | 10/135 (7.4) | 0.067 |
| Presentation with DKA, n (%) | 41/121 (34) | 15/43 (35) | 0.906 | 20/120 (16.7) | 17/125 (13.6) | 0.503 |
| FPG at presentation, mg/dL, median (IQR) | 220 (139) | 240 (189) | 0.671 | 212 (142) | 210 (171) | 0.966 |
| HbA1c at presentation, %, median (IQR) | 8.4 (2.2) | 8.6 (3.3) | 0.363 | 8.5 (3.1) | 8.8 (3.2) | 0.161 |
| Insulin dose at baseline, IU/kg/d, median (IQR) | 0.37 (0.2) | 0.33 (0.3) | 0.444 | 0.31 (0.2) | 0.29 (0.2) | ***0.041*** |
| C-peptide, ng/mL, median (IQR) | 0.02 (0.14) | 0.36 (0.94) | 0.057 | 0.2 (0.7) | 0.77 (1.2) | ***<0.001*** |
| eGFR, mL/min/1.73 m^2^, median (IQR) | 125 (21) | 129 (11) | 0.226 | 115 (39) | 117 (25) | 0.316 |
| Total-C, mg/dL, median (IQR) | 168 (50) | 160 (43) | 0.072 | 172 (48) | 168 (46) | 0.464 |
| LDL-C, mg/dL, median (IQR) | 100 (44) | 98.5 (38) | 0.269 | 100 (44) | 98.5 (37) | 0.711 |
| HDL-C, mg/dL, median (IQR) | 55 (17) | 50.5 (19) | 0.096 | 49 (22) | 50.5 (21) | 0.762 |
| TG, mg/dL, median (IQR) | 74 (46) | 78 (61) | 0.709 | 98 (80) | 79 (56) | 0.063 |
| Follow-up | | | | | | |
| Other autoimmune diseases, n (%) | 57/134 (42.5) | 21/47 (44.7) | 0.798 | 50/117 (42.7) | 48/121 (39.7) | 0.631 |
| Hypertension, n (%) | 31 (20.5) | 6 (12) | 0.177 | 33 (22) | 25 (18) | 0.363 |
| Dyslipidemia/hyperlipidemia, n (%) | 33 (22) | 7 (14) | 0.228 | 52 (35) | 41 (29) | 0.271 |
| Overweight, n (%) | 15/58 (26) | 4/21 (19) | 0.531 | 39/89 (44) | 27/60 (45) | 0.887 |
| Obesity, n (%) | 1/64 (1.6) | 1/22 (4.5) | 0.423 | 17/97 (17.5) | 13/72 (18) | 0.929 |
| Any macrovascular complications, n (%) | 9 (6) | 1 (2) | 0.264 | 11 (7.4) | 12 (8.6) | 0.709 |
| CAD, n (%) | 4 (2.7) | 0 | 0.243 | 7 (4.7) | 8 (5.7) | 0.707 |
| CVA, n (%) | 2 (1.3) | 0 | 0.413 | 1 (0.7) | 1 (0.7) | 0.969 |
| PAD, n (%) | 1 (0.7) | 0 | 0.564 | 2 (1.4) | 2 (1.4) | 0.950 |
| DFU, n (%) | 4 (2) | 1 (2) | 0.798 | 4 (2.7) | 2 (1.4) | 0.449 |
| Any microvascular complications, n (%) | 56 (37) | 19 (38) | 0.908 | 53 (35.6) | 45 (32) | 0.538 |
| Retinopathy, n (%) | 36 (24) | 5 (10) | ***0.034*** | 29 (20.7) | 14 (10) | ***0.013*** |
| Neuropathy, n (%) | 26 (17) | 12 (24) | 0.298 | 29 (20.7) | 26 (18.7) | 0.695 |
| UACR >30 mg/g, n (%) | 37/147 (25.2) | 13 (26) | 0.907 | 22/137 (16) | 27/137 (19.7) | 0.431 |
| eGFR <60 mL/min/1.73 m^2^, n (%) | 9/133 (6.8) | 0 | 0.062 | 10/126 (8) | 3/130 (2.3) | ***0.040*** |

SD, standard deviation; IQR, interquartile range; T1DM, type 1 diabetes mellitus; T2DM, type 2 diabetes mellitus; BMI, body mass index; DKA, diabetic ketoacidosis; FPG, fasting plasma glucose; HbA1c, glycated hemoglobin A1c; eGFR, estimated glomerular filtration rate; Total-C, total cholesterol; LDL-C, low-density lipoprotein cholesterol; HDL-C, high-density lipoprotein cholesterol; TG, triglycerides; CAD, coronary artery disease; CVA, cerebrovascular accident; PAD, peripheral artery disease; DFU, diabetic foot ulcer; UACR, urine albumin-to-creatinine ratio.

**Suppl. Table 3.** Comparison of clinical and laboratory findings according to age-at-onset status in patients who developed microvascular complications

|  | Microvascular complications | | |
| --- | --- | --- | --- |
| Variables | **child-onset (n=75)** | **adult-onset (n=98)** | **p-value** |
| Age, years, mean±SD | 34.9±8.7 | 48.5±13.2 | ***<0.001*** |
| Gender, female, n (%) | 42 (56) | 55 (56) | 0.987 |
| DM duration, years, median (IQR) (range) | 24 (11) (8-47) | 19 (16) (2-45) | ***0.001*** |
| Follow-up duration, years, median (IQR) | 5 (5) | 7 (8) | ***0.004*** |
| Family history, n (%) | 49/74 (66) | 63/91 (69) | 0.680 |
| T1DM | 16/74 (21.6) | 18/91 (19.8) | 0.771 |
| T2DM | 37/74 (50) | 49/91 (53.8) | 0.623 |
| BMI, kg/m^2^, mean±SD | 22.6±3.4 | 25.7±4.3 | ***0.001*** |
| Overweight, n (%) | 7/26 (27) | 27/53 (51) | ***0.043*** |
| Obese, n (%) | 1/28 (3.6) | 15/59 (25.4) | ***0.014*** |
| Smoking history, n (%) | 14/67 (21) | 28/82 (34) | 0.074 |
| Alcohol, n (%) | 6/73 (8.2) | 9/94 (9.6) | 0.761 |
| Presentation with DKA, n (%) | 22/60 (36.7) | 11/79 (14) | ***0.002*** |
| IDA-HbA1c >8.9% at presentation, n (%) | 40/62 (64.5) | 68/82 (83) | ***0.012*** |
| C-peptide levels<0,383 at presentation, n (%) | 22/24 (92) | 22/37 (59.5) | ***0.006*** |
| Hypertension, n (%) | 32 (42.7) | 40 (40.8) | 0.807 |
| Hyperlipidemia, n (%) | 24 (32) | 46 (47) | ***0.047*** |
| Macrovascular complications, n (%) | 8 (10.7) | 18 (18.4) | 0.160 |
| Coronary heart disease, n (%) | 3 (4) | 11 (11) | 0.089 |
| Cerebrovascular accident, n (%) | 2 (2.7) | 1 (1) | 0.411 |
| Peripheral artery disease, n (%) | 1 (1.3) | 4 (4.1) | 0.280 |
| Diabetic foot ulcer, n (%) | 4 (5.3) | 6 (6) | 0.826 |
| Microvascular complications, n (%) | NA | NA |  |
| Retinopathy, n (%) | 41 (55) | 42 (43) | 0.139 |
| Neuropathy, n (%) | 38 (50.7) | 53 (55) | 0.605 |
| UACR >30 mg/g, n (%) | 50 (67) | 49 (50.5) | 0.025 |
| eGFR <60 mL/min/1.73 m2, n (%) | 9/71 (12.7) | 13/90 (14.4) | 0.746 |
| At least one islet autoantibody positivity, n (%) | 9/16 (56.3) | 33/44 (77) | 0.122 |
| GADA, n (%) | 5/16 (31.3) | 28/44 (63.6) | ***0.026*** |
| ICA, n (%) | 5/16 (31.3) | 17/41 (41.5) | 0.477 |
| Autoimmune disease, n (%) | 30/69 (43.5) | 39/82 (47.6) | 0.616 |
| Insulin dose at baseline, IU/kg/d, median (IQR) | 0.32 (0.2) | 0.3 (0.2) | 0.722 |
| FPG at presentation, mg/dL, median (IQR) | 207 (177) | 231 (184) | 0.243 |
| HbA1c at presentation, %, median (IQR) | 8.3 (1.9) | 9.2 (3) | ***0.002*** |
| C-peptide, ng/mL, median (IQR) | 0.016 (0.24) | 0.094 (0.7) | 0.051 |
| eGFR, ml/min/1.73 m2, median (IQR) | 120 (46) | 113 (39) | 0.480 |
| Total-C, mg/dL, median (IQR) | 178 (52) | 177 (55) | 0.766 |
| LDL-C, mg/dL, median (IQR) | 104 (49) | 102 (42) | 0.768 |
| HDL-C, mg/dL, median (IQR) | 58 (18) | 50 (23) | ***0.018*** |
| TG, mg/dL, median (IQR) | 81 (70) | 102.5 (80) | ***0.015*** |

SD, standard deviation; BMI, body mass index; T1DM, type 1 diabetes mellitus; T2DM, type 2 diabetes mellitus; DKA, diabetic ketoacidosis; GADA, glutamic acid decarboxylase autoantibody; ICA, islet cell cytoplasmic autoantibody; HbA1c, glycated hemoglobin A1c; FPG, fasting plasma glucose; IQR, interquartile range; eGFR, estimated glomerular filtration rate; Total-C, total cholesterol; LDL-C, low-density lipoprotein cholesterol; HDL-C, high-density lipoprotein cholesterol; TG, triglycerides. UACR, urine albumin-to-creatinine ratio; eGFR, estimated glomerular filtration rate

**Supplementary table 4:** Comparison of clinical and laboratory findings according to age at onset status in patients who developed macrovascular complications

|  | Macrovascular complications | | |
| --- | --- | --- | --- |
| Variables | **child-onset (n=10)** | **adult-onset (n=23)** | **p value** |
| Age, years, mean±SD | 41.9±9.5 | 54.8±13.7 | ***0.011*** |
| Gender, female, n (%) | 4 (40) | 11 (48) | 0.678 |
| DM duration, years, median (IQR) (range) | 29.5 (16) (13-47) | 22 (14) (5-36) | ***0.016*** |
| Follow-up duration, years, median (IQR) | 5 (5) | 7.5 (8) | 0.127 |
| Family history, n (%) | 7 (70) | 18/20 (90) | 0.166 |
| T1DM | 4 (40) | 4/20 (20) | 0.243 |
| T2DM | 4 (40) | 15/20 (75) | 0.061 |
| BMI, kg/m^2^, mean±SD | 21.4±3 | 25.6±2.8 | ***0.034*** |
| Overweight, n (%) | 0/2 | 5/10 (50) | 0.190 |
| Obese, n (%) | 0/3 | 2/15 (13.3) | 0.502 |
| Smoking history, n (%) | 3/8 (37.5) | 5/16 (31.3) | 0.759 |
| Alcohol, n (%) | 1/9 (11) | 3/21 (14.3) | 0.815 |
| Presentation with DKA, n (%) | 1/6 (16.7) | 0/14 | 0.117 |
| IDA-HbA1c >8.9% at presentation, n (%) | 4/8 (50) | 14/17 (82.4) | 0.093 |
| C-peptide levels<0,383 at presentation, n (%) | 2/2 (100) | 4/5 (80) | 0.495 |
| Hypertension, n (%) | 7 (70) | 14 (61) | 0.616 |
| Hyperlipidemia, n (%) | 6 (60) | 17 (74) | 0.424 |
| Macrovascular complications, n (%) | NA | NA |  |
| Coronary heart disease, n (%) | 4 (40) | 15 (65) | 0.282 |
| Cerebrovascular accident, n (%) | 2 (20) | 2 (8.7) | 0.361 |
| Peripheral artery disease, n (%) | 1 (10) | 4 (18) | 0.555 |
| Diabetic foot ulcer, n (%) | 5 (50) | 6 (26) | 0.181 |
| Microvascular complications, n (%) | 8 (80) | 18 (78.3) | 0.911 |
| Retinopathy, n (%) | 5 (50) | 12 (52) | 0.909 |
| Neuropathy, n (%) | 7 (70) | 16 (70) | 0.980 |
| UACR >30 mg/g, n (%) | 3 (30) | 8 (35) | 0.919 |
| eGFR <60 mL/min/1.73 m^2^, n (%) | 1 (10) | 5/19 (26.3) | 0.303 |
| At least one islet autoantibody positivity, n (%) | 0/1 | 11/12 (92) | ***0.015*** |
| GADA, n (%) | 0/1 | 9/11 (82) | 0.070 |
| ICA, n (%) | 0/1 | 9/11 (82) | 0.070 |
| Autoimmune disease, n (%) | 2/9 (22) | 8/19 (42) | 0.305 |
| Insulin dose at baseline, IU/kg/d, median (IQR) | 0.25 (0.2) | 0.28 (0.14) | 0.914 |
| FPG at presentation, mg/dL, median (IQR) | 211 (123) | 250 (177) | 0.186 |
| HbA1c at presentation, %, median (IQR) | 7.9 (3.1) | 8.8 (1.8) | 0.218 |
| C-peptide, ng/mL, median (IQR) | 0.016 (0.08) | 0.14 (0.9) | 0.190 |
| eGFR, ml/min/1.73 m^2^, median (IQR) | 70 (51) | 82.3 (85) | 1.000 |
| Total-C, mg/dL, median (IQR) | 193 (32) | 174 (49) | 0.212 |
| LDL-C, mg/dL, median (IQR) | 126 (41) | 105.5 (42) | 0.121 |
| HDL-C, mg/dL, median (IQR) | 60 (36) | 56 (15) | 0.597 |
| TG, mg/dL, median (IQR) | 91 (107) | 74 (103) | 0.749 |

SD, standard deviation; IQR, interquartile range; T1DM, type 1 diabetes mellitus; T2DM, type 2 diabetes mellitus; BMI, body mass index; DKA, diabetic ketoacidosis; GADA, glutamic acid decarboxylase autoantibody; ICA, islet cell cytoplasmic autoantibody; FPG, fasting plasma glucose; HbA1c, glycated hemoglobin A1c; IDA-HbA1c, insulin dose-adjusted HbA1c; eGFR, estimated glomerular filtration rate; Total-C, total cholesterol; LDL-C, low-density lipoprotein cholesterol; HDL-C, high-density lipoprotein cholesterol; TG, triglycerides; CAD, coronary artery disease; CVA, cerebrovascular accident; PAD, peripheral artery diasease; DFU, diabetic foot ulcer; UACR, urine albumin-to-creatinine ratio.
